# Towards Splicing Therapy for Lysosomal Storage Disorders: Methylxanthines and Luteolin Ameliorate Splicing Defects in Aspartylglucosaminuria and Classic Late Infantile Neuronal Ceroid Lipofuscinosis

**DOI:** 10.3390/cells10112813

**Published:** 2021-10-20

**Authors:** Antje Banning, Ritva Tikkanen

**Affiliations:** Institute of Biochemistry, Medical Faculty, University of Giessen, Friedrichstrasse 24, 35392 Giessen, Germany; Antje.Banning@biochemie.med.uni-giessen.de

**Keywords:** RNA splicing, splice-modulators, xanthines, luteolin, caffeine, splicing defects, genetic diseases

## Abstract

Splicing defects caused by mutations in the consensus sequences at the borders of introns and exons are common in human diseases. Such defects frequently result in a complete loss of function of the protein in question. Therapy approaches based on antisense oligonucleotides for specific gene mutations have been developed in the past, but they are very expensive and require invasive, life-long administration. Thus, modulation of splicing by means of small molecules is of great interest for the therapy of genetic diseases resulting from splice-site mutations. Using minigene approaches and patient cells, we here show that methylxanthine derivatives and the food-derived flavonoid luteolin are able to enhance the correct splicing of the AGA mRNA with a splice-site mutation c.128-2A>G in aspartylglucosaminuria, and result in increased AGA enzyme activity in patient cells. Furthermore, we also show that one of the most common disease causing *TPP1* gene variants in classic late infantile neuronal ceroid lipofuscinosis may also be amenable to splicing modulation using similar substances. Therefore, our data suggest that splice-modulation with small molecules may be a valid therapy option for lysosomal storage disorders.

## 1. Introduction

Aspartylglucosaminuria (AGU, OMIM 208400) is a rare lysosomal storage disorder that is caused by a deficiency of the aspartylglucosaminidase (AGA; N4-(β-N-acetylglucosaminyl)-L-asparaginase, EC 3.5.1.26) enzyme. AGU manifests mainly as a progressive loss of mental capabilities and increasing physical deterioration. For a recent review on AGU disease pathogenesis, the readers are encouraged to consult [1,2]. The highest number of AGU patients is found in Finland, where almost all AGU patients are homo- or heterozygous for the *AGA* gene variant known as AGU_FIN-major_. However, patients of non-Finnish origin usually exhibit their own family-specific variants that may be compound heterozygous, or homozygous in the case of consanguinity. The spectrum of disease-causing *AGA* gene variants is wide, ranging from deletions and insertions to missense and nonsense point mutations [3].

Currently, an approved therapy for AGU is not available, despite research efforts that have been dedicated to preclinical studies. Optimally, a therapy for AGU should be available for as many patients as possible. Due to the fact that the AGU_FIN-major_ variant is found in almost all Finnish AGU patients, it represents an attractive target for therapy approaches. We have focused our previous efforts on repurposing of small molecules for the treatment of AGU. We recently showed that the AGU_FIN-major_ disease variant with the amino acid substitutions Arg161Gln and Cys163Ser is amenable to therapy with a pharmacological chaperone, tri-methyl-glycine (betaine, trade name Cystadane) [4], and a clinical trial with this substance is currently running in Finland [5]. However, not all AGU disease variants, including some of the missense variants, are treatable with this substance, as its effect is based on correction of the protein folding of the mutated enzyme [4]. Therefore, further therapy options are required for patients that do not exhibit the AGU_FIN-major_ missense variant, or have, e.g., nonsense variants. We recently described a potential read-through therapy with Amlexanox for an AGU patient with a compound heterozygous nonsense variant [6]. In addition, we are involved in developing a gene therapy for AGU [7].

Among the most difficult-to-treat gene variants in terms of small molecule therapy are those that result in splicing defects. About 9–15% of all known human disease-causing variants affect the consensus splice-sites [8,9]. Most of the previous therapy approaches that target disease-causing splicing variants are based on antisense oligonucleotides (ASOs) that mask splicing sites and produce alternatively spliced protein variants that may be only partially functional. In Duchenne muscular dystrophy (DMD) that is caused by mutations in the gene encoding for the dystrophin protein, pathogenic gene variants are often found in exon 51, and an ASO that causes skipping of the mutated exon and results in a partly functional dystrophin protein has produced clinical improvements in DMD patients [10,11,12,13]. Similarly, Nusinersen (Spinraza) is a therapeutic oligonucleotide used in spinal muscular atrophy (SMA) to induce alternative splicing of the *SMN2* (*survival motor neuron 2*) gene, a paralog of *SMN1* that is deficient in SMA. Nusinersen treatment results in inclusion of an additional exon and expression of an alternative isoform of SMN2 protein that can partially compensate for the loss of function of SMN1 [14,15]. Very recently, splicing modifying ASOs were also described for the *CLN3* (*ceroid-lipofuscinosis*, *neuronal 3*) gene that is deficient in the juvenile form of neuronal ceroid lipofuscinosis (JNCL) [16]. Most JNCL patients exhibit a genomic deletion that encompasses exons 7 and 8 of the *CLN3* gene. The splice-modulating ASOs target exon 5 that is also excluded, resulting in a partially functional, truncated CLN3 protein due to correction of the frame-shift produced by exon 7 and 8 exclusion. In another NCL disease, an antisense splice-modulator known as Milasen for the *CLN7* gene was developed for a single patient exhibiting an intronic retrotransposon [17]. A novel exon is thus included in the mRNA, producing a frame-shifted protein variant. Milasen masks the aberrant splice-site in the retrotransposon and thus corrects the splicing [17]. These examples show that ASOs have significant potential for the treatment of genetic diseases involving splice variants with additional splice-sites that can be masked by the ASOs. However, they are extremely expensive to develop and to use, and they require a life-long, invasive administration of the therapeutic ASO. Furthermore, ASOs are of limited use for splice variants that result from a loss of a splice-site.

Due to the above reasons, modulation or correction of splicing using small molecules that are orally available would be a great advantage in the treatment of diseases that result from aberrant splicing. Very recently, small, orally available molecules, such as Risdiplam, Branaplam and TEC-1 that are capable of altering *SMA2* splicing in a similar manner as Nusinersen, have been described and are currently tested in clinical trials [18,19,20,21]. The first encouraging results suggest that Risdiplam shows clinical efficacy in infants with type 1 SMA. Encouragingly, in humans, it does not seem to exhibit toxic effects on the retina that were observed in animal studies [18,22]. As compared to Risdiplam, TEC-1 appears to show a higher specificity towards *SMA2*, with less effect on the splicing of secondary splice targets such as forkhead box protein M1 (*FOXM1*), huntingtin (*HTT*) and galactosylceramidase (*GALC*), which may be an advantage over Risdiplam in clinical use, resulting in less potential side effects [20].

In many genetic diseases, splice consensus site mutations are frequent, whereas only few splice-site variants have been detected in AGU. Here, we describe a female AGU patient with a homozygous point mutation in the 3′ splice-acceptor site of intron 1, at the border of exon 2. We show that the splicing defect associated with this gene variant can be ameliorated with natural substances that enhance splicing. Furthermore, these substances are also capable of improving the splicing of a common *TPP1* gene variant that is associated with another lysosomal storage disorder. Thus, our data suggest that splicing enhancement using small molecules may be a potential treatment for lysosomal storage disorders associated with disease-causing splice variants.

## 2. Materials and Methods

### 2.1. Cell Culture

HEK293T (human embryonic kidney) cells were cultured in Dulbecco’s modified Eagle’s medium (DMEM) with high glucose, 10% fetal calf serum (FCS), 1% penicillin/streptomycin (all from Gibco, Thermo Fisher Scientific, Schwerte, Germany). AGA knockout HEK293T cells without endogenous AGA expression were created by CRISPR/Cas9 and have been described before [23]. Primary skin fibroblasts carrying the homozygous intronic mutation c.128-2A>G in the *AGA* gene were obtained from a 7-year-old female Australian patient of Indian heritage at Murdoch Children’s Research Institute (Melbourne, Victoria, Australia) and have been described before [6]. The parents provided a signed informed consent for the biopsy, fibroblasts culture, storage, enzymatic and molecular analyses, and the procedures were approved by the Murdoch Children’s Research Institute (Melbourne, Victoria, Australia). Immortalized, hTERT-transformed normal human skin fibroblasts were used as control [4]. All fibroblasts were cultured in DMEM (high glucose), 10% FCS, 1% penicillin/streptomycin, 1% non-essential amino acids and 1% sodium pyruvate. All cells were grown at 8% CO_2_ and 37 °C.

### 2.2. Minigene Constructs and Transfection

The *AGA*-minigene-pcDNA3 and *AGA*-minigene-Fluc-pcDNA3 constructs contain approximately 2900 bp of PCR-amplified *AGA* genomic sequence spanning exons 1–3 with the mutation c.128-2A>G, either containing the firefly luciferase coding sequence at the 3′ end or without luciferase. The *TPP1*-minigene-pcDNA3 constructs with or without luciferase contain approximately 800 bp of *TPP1* genomic sequence spanning exons 5–7 with the mutation c.509-1G>C. The firefly luciferase coding sequence (Fluc) was PCR amplified from pGL3-basic (Promega, Walldorf, Germany; GenBank number U47295) without the ATG start codon and was cloned in-frame 3′ of the *AGA* exon 3 or *TPP1* exon 7, respectively. All constructs were verified by DNA sequencing (Seqlab, Göttingen, Germany). Primer sequences for cloning are shown in Table 1.

For transient transfections, cells were seeded onto 12-well plates on the day before transfection. For transfections, 400 ng of expression plasmid DNA and 50 ng of pRL-TK Renilla luciferase control reporter vector (Promega, Walldorf, Germany) were transfected using MACSfectin™ (Miltenyi Biotec, Bergisch Gladbach, Germany) according to the manufacturer’s protocol. Fibroblasts were transfected with Neon Transfection System (Invitrogen, Karlsruhe, Germany). The following day, the cells were transferred onto 24-well plates, treated with specific substances and harvested after 24 h of treatment in lysis juice for firefly and renilla luciferase assay (PJK GmbH, Kleinblittersdorf, Germany). Determination of firefly and renilla luciferase activity was performed with a Tecan infinite M200 plate reader using 20 µL of lysate and 85 µL of beetle or renilla juice (PJK) as reagents. Relative luciferase activity was calculated by dividing the firefly luciferase activity by the renilla luciferase activity.

### 2.3. Treatment of Patient Fibroblasts with Recombinant AGA or Splicing Modulating Compounds

Fibroblasts were seeded into 6-well plates and treated with the following compounds: Caffeine, theophylline, theobromine, pentoxifylline, doxofylline (1–7.5 mM, solvent H_2_O; TCI, Eschborn, Germany), luteolin (10–50 µM, solvent DMSO; Biozol, Eching, Germany) or kinetin (10–100 µM, solvent DMSO; TCI, Eschborn, Germany) for 24 h (RNA) or 48 h (protein lysate). Recombinant Strep-tagged AGA was purified from cell culture medium of HEK293T cells overexpressing AGA as described in Banning et al. 2016 [4]. Quantification of purified proteins was done densitometrically in SDS-PAGE with BSA as reference. Cells were harvested in lysis buffer (50 mM Tris pH 7.4, 150 mM NaCl, 2 mM EDTA, 1% NP-40), supplemented with protease inhibitor cocktail (Sigma-Aldrich, Taufkirchen, Germany). Protein concentrations in the lysates were determined according to the Bradford method.

### 2.4. Antibodies

Mouse monoclonal antibodies against firefly luciferase (sc-74548) and U2AF65 (sc-53942) were from Santa Cruz Biotechnology (Heidelberg, Germany). A mouse monoclonal antibody against GAPDH and rabbit monoclonal antibodies against SF3B1 (ab170854) and SRSF2 (SC35, ab204916) were purchased from Abcam (Cambridge, UK). A mouse monoclonal antibody against Streptag II was from Novagen^®^ (Sigma-Aldrich, Taufkirchen, Germany).

### 2.5. Enzyme Activity Measurements

In most experiments, AGA activity was measured fluorometrically as described [4]. Reaction mixtures contained 10–20 µL cell lysate or lysis buffer only for a blank and 20 µL of Asp-AMC [L-Aspartic acid β-(7-amido-4-methylcoumarin)], 50 µM in McIlvain’s phosphate-citrate buffer pH 6.5. The samples were incubated for 24 h at 37 °C, after which the reaction was stopped by addition of 200 µL McIlvain’s buffer pH 4.5. Triplicate samples were measured with a Tecan infinite M200 plate reader (355 nm excitation and 450 nm emission). AGA activity in samples that had been treated with luteolin were measured photometrically with the Morgan–Elson-assay using 2-acetamido-1-ß-(L-aspartamido)-1,2-dideoxy-ß-D-glucose (AADG, Santa Cruz Biotechnology) as substrate, because of the fluorescence quenching properties of luteolin. In short, 12.5 µL of cell lysate were mixed with 100 nmol AADG, 3.35 µL 0.5 M KH_2_PO_4_, pH 8 and 0.9% NaCl to reach a total volume of 25 µL. Samples were incubated at 37 °C for 24 h. Reactions were stopped by adding 53.5 µL of 0.8 M borate buffer pH 9.1 and boiling for 5 min at 100 °C. Five volumes of 4-(Dimethylamino)benzaldehyde (DMAB) reagent (10 mg DMAB/mL glacial acetic acid) were added and samples incubated for 20 min at 37 °C until a purple colour was visible. The liberated N-acetylglucosamine was measured at 585 nm. N-acetylglucosamine (25–1000 µM) was used as standard.

### 2.6. Quantitative Real-Time PCR, Standard PCR and Sequencing of PCR Products

For qPCRs, total RNA was isolated with peqGOLD TriFast™ (VWR, Darmstadt, Germany) and a DNAse step was included. Total RNA (1–3 µg) were reverse transcribed with 150 fmol oligo(dT) primers and M-MuLV reverse transcriptase (NEB, Frankfurt, Germany) in a total volume of 45 µL. Real-time quantitative PCRs were performed using the CFX Connect Real-Time PCR Detection System (Bio-Rad, Munich, Germany). Annealing temperature was 60 °C for all primers. The reactions were done as duplicates with 0.8 µL cDNA in a total volume of 10 µL using iTaq^TM^ Universal SYBR Green Supermix (Bio-Rad, Munich, Germany). PCR products were quantified with the ∆Ct-method. For normalization, the mean of the reference genes *B2M*, *Rpl13a*, *Ywhaz* and *TBP* was used. PCRs to confirm the correct fusion of *AGA* exons 1 and 2 were carried out the same way, but with Q5 polymerase (New England Biolabs, Frankfurt, Germany) instead of the SYBR Green Supermix. PCR samples were run on agarose gels, and the resulting bands were gel-purified and sequenced (Seqlab, Göttingen, Germany). The primer sequences are shown in Table 2.

### 2.7. Immunofluorescence

Cells were seeded on glass coverslips and cultured for at least 2 days. For staining of acidic cell compartments, the cells were incubated with 2.5 µM Lysotracker-Red (Invitrogen) in cell culture medium for 30 min. All cells were fixed in 4% paraformaldehyde for 10 min at room temperature, washed and embedded in ROTI^®^Mount FluorCare DAPI (Carl Roth, Karlsruhe, Germany). The samples were analyzed with a Zeiss LSM710 Confocal Laser Scanning Microscope (Carl Zeiss, Oberkochen, Germany).

### 2.8. SRSF1 and SRSF2 Overexpression

The coding sequences of the splice factors arginine rich splicing factors 1 and 2 (SRSF1 and SRSF2) were PCR amplified from HEK293T cDNA and cloned by XbaI and BamHI digestion into pEXPR-IBA103 (IBA, Göttingen, Germany) with a C-terminal Twin-Strep-tag. The primer sequences were: SRSF1-XbaI fwd: 5′- CTATATCTAGAATGTCGGGAGGTGGTGTGATTC-3′, SRSF1-nonstop-BamHI rev: 5′- CTATAGGATCCTGTACGAGAGCGAGATCTGCTA-3′ SRSF2-XbaI fwd: 5′- CTATATCTAGAATGAGCTACGGCCGCCCCCCTC-3′, SRSF2-nonstop-BamHI rev: 5′-CTATAGGATCCAGAGGACACCGCTCCTTCCTCTTC-3′. All constructs were verified by DNA sequencing (Seqlab, Göttingen, Germany). Transient transfections were performed as described above. 200 ng of expression plasmid DNA and 200 ng of the AGA minigene construct were cotransfected. For reporter gene assays, this was done together with 50 ng of pRL-TK Renilla luciferase control reporter vector (Promega, Walldorf, Germany). All further steps were as described in Section 2.2.

### 2.9. Statistical Analysis

All experiments were performed at least three times. Data are expressed as mean ± SD. Statistical comparisons between groups were made using Student’s *t*-tests, one-way or two-way ANOVA (Analysis of Variance), as appropriate, using the GraphPad Prism 5 software (San Diego, CA, USA). Values of *p* < 0.05 were considered significant (*) while values of *p* < 0.01 were considered very significant (**) and *p* < 0.001 extremely significant (***).

## 3. Results

### 3.1. An AGU Patient with a Homozygous Splice Acceptor Site Mutation

**Figure 1:**
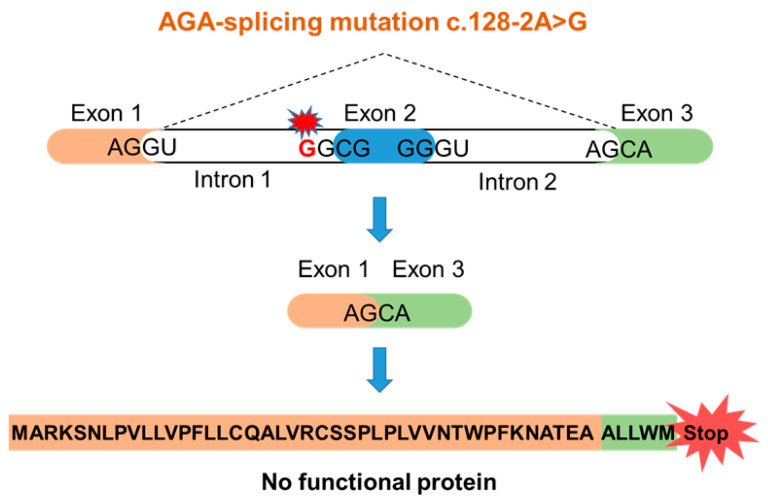
Predicted consequences of the c.128-2A>G AGU mutation.

A seven-year-old female of Indian origin with a developmental delay was presented at Murdoch Children’s Research Institute (Melbourne, Victoria, Australia) [6]. Metabolic analysis of the urine showed a substantial increase of glycoasparagines (mainly aspartylglucosamine), suggesting AGU as the underlying disease. Sequence analysis of the *AGA* gene revealed a homozygous point mutation at the 3′-splice-acceptor site of the intron 1 (c.128-2A>G, sequencing data in Supplementary Figure S1). The consensus splice motif AG at the end of the intron 1 is exchanged into GG. Therefore, it can be predicted that exon 2 is spliced out together with introns 1 and 2, resulting in a frame-shift and an early translation termination (Figure 1). This variant can be classified as a potentially harmful one, but it has not been reported in an AGU patient before. The parents of the patient are not aware of being related with each other, suggesting a non-consanguineous origin of the mutated alleles.

**Figure 2:**
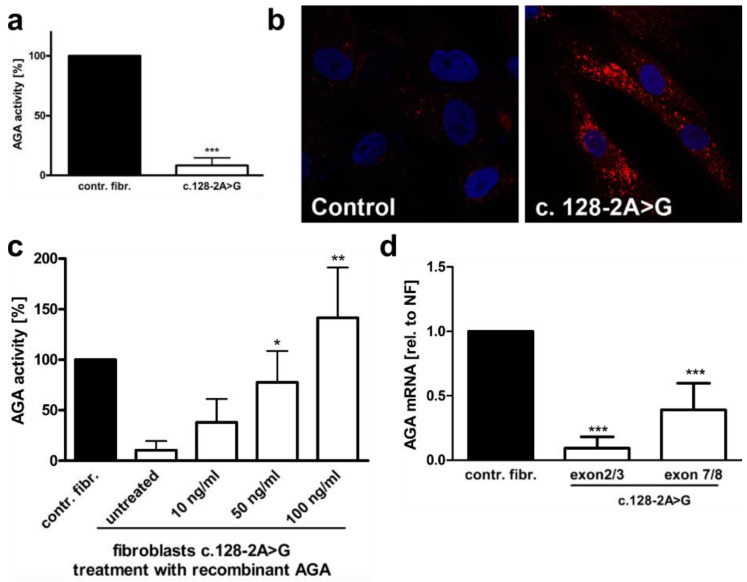
Characterization of the c.128-2A>G AGU variant. (**a**) Control and c.128-2>G AGU fibroblasts were lysed, and the AGA activity was measured fluorometrically. Eleven independent experiments were performed. The graph shows the mean of the data ± SD. Statistical analysis was performed with unpaired t test. (**b**) Control fibroblast and the fibroblasts of the patient were stained with Lysotracker-red to visualize lysosomal abnormalities. (**c**) The patient fibroblasts were treated with the indicated amounts of recombinantly expressed, Strep-tagged AGA fusion protein for 48 h, and the AGA enzyme activity was measured and compared with the control fibroblasts whose activity was set to 100%. (**d**) Quantitative real-time PCR of *AGA* mRNA in patient fibroblasts with the c.128-2A>G variant. *AGA* transcript was detected with two different primer pairs, one for exons 2 and 3 and the other one for exons 7 and 8. The c.128-2A>G variant leads to skipping of exon 2, as indicated by the lower transcript amount with the exons 2+3 primers as compared to the primer pair for exons 7+8. Bars represent the mean ± SD of 10 independent experiments. Statistical analysis was done by one-way ANOVA against the control fibroblasts. Values of *p* < 0.05 were considered significant (*) while values of *p* < 0.01 were considered very significant (**) and *p* < 0.001 extremely significant (***).

A skin biopsy of the patient was performed, and fibroblast cultures were established for further analysis. The fibroblasts have been described in a previous study of ours [6]. The AGA enzyme activity in the fibroblasts cultures was 8.3% (SD 6.3%) of control fibroblasts (Figure 2a), consistent with AGU. Lysotracker staining of the fibroblasts revealed an enlarged lysosomal compartment, typical of lysosomal storage disorders (Figure 2b). Treatment of the patient fibroblasts with recombinant, purified AGA [4] resulted in normalization of the AGA enzyme activity (Figure 2c). A qRT-PCR with primer pairs for *AGA* exons 2 and 3 or 7 and 8 showed a reduction of the *AGA* mRNA in the patient cells (Figure 2d). However, while the primer pair for exons 7 and 8 suggested a reduction to 39.0% of the mRNA level in control fibroblasts, the reduction observed with the primer pair for exons 2 and 3 was more pronounced (down to 9.4% of the control), suggesting that a splicing defect of exons 2 and/or 3 might be present.

**Figure 3:**
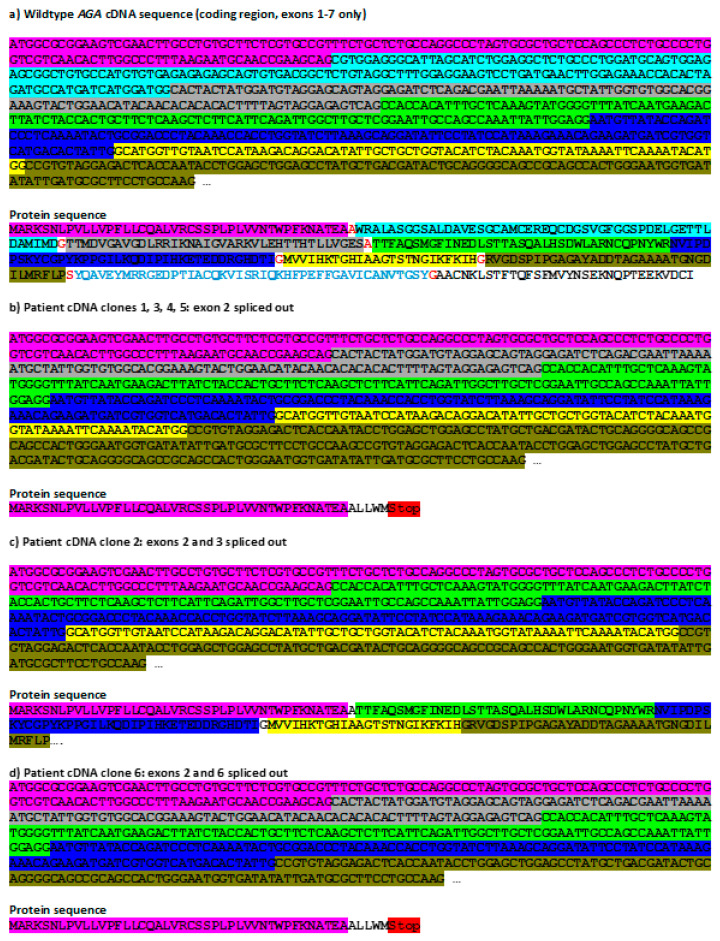
Sequence analysis of cloned AGA cDNAs from patient fibroblasts with the c.128-2A>G variant. (**a**) *AGA* reference coding sequence (NM_000027.4) and translation thereof. Individual exons are shown in different colors. The codons for the amino acids in red (wildtype protein sequence) at the borders of two exons contain bases from both exons. (**b**–**d**) Total RNA was isolated from patient fibroblasts, reversely transcribed into cDNA, and the *AGA* coding region was amplified by standard PCR. The products were cloned into pcDNA3 and six clones were sequenced. In all cases, exon 2 was missing, either alone (**b**) or in combination with exon 3 (**c**) or exon 6 (**d**). Mis-splicing leads either to introduction of an early stop codon (**b**,**d**) or an in-frame deletion of 89 amino acids (**c**).

Since the above data are suggestive of a splicing defect, we characterized the *AGA* splicing variants in the patient cells. The *AGA* open reading frame was PCR-amplified from a cDNA as described in [4], cloned into a plasmid vector, and six cDNA clones were sequenced (Figure 3). The WT sequence and the resulting protein translation are shown in Figure 3a. The most abundant (four out of six clones analyzed) *AGA* splicing isoform in the patient cells exhibits an exclusion of the exon 2 (Figure 3b), as expected due to the mutation in the acceptor splice-site preceding exon 2. In addition, single clones that contained either a deletion of exons 2 and 3 (Figure 3c) or exons 2 and 6 (Figure 3d) were also detected. Clones with out-of-frame deletions of exon 2 or exons 2+6 result in an early STOP codon and a predicted highly truncated protein. However, a deletion of exons 2 and 3 results in normalization of the reading frame from exon 4 onwards, and the predicted protein would exhibit a deletion of 89 amino acids in the N-terminal half of the α subunit, but otherwise a normal amino acid sequence.

### 3.2. A Luciferase Minigene Assay for Testing Substances That Affect AGA Splicing

**Figure 4:**
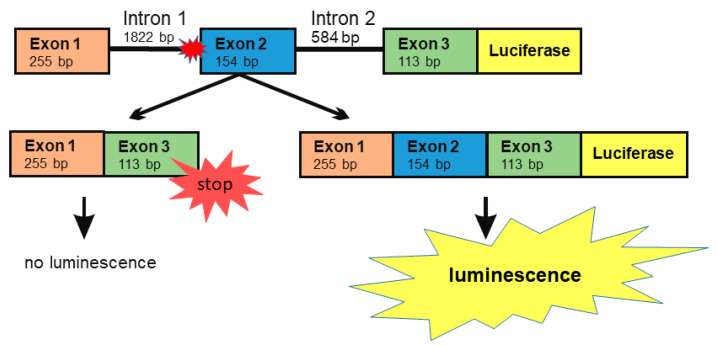
The *AGA* minigene constructs with firefly luciferase. Genomic DNA was isolated from the patient fibroblasts. The region comprising exons 1–3 was amplified by standard PCR and cloned into the pcDNA3 vector. Firefly luciferase (without the ATG start codon) was cloned in-frame downstream of the *AGA* minigene. Skipping of exon 2 will lead to a frame shift and early termination of translation, resulting in lack of luciferase signal. The correct splicing with inclusion of all three exons produces a fusion protein containing a C-terminal luciferase protein whose activity can be measured by chemiluminescence.

Numerous natural substances exhibit potential as splicing enhancers due to their capability to enhance mRNA stability or to affect splicing directly (for review, see [24]). For testing such substances, we developed a minigene assay that is based on a DNA construct containing *AGA* exons 1, 2 and 3, together with the introns 1 and 2, followed by the firefly luciferase coding sequence (Figure 4). This construct contains the splice acceptor site mutation -2A>G in intron 1. If exon 2 is spliced out, altered reading frame in exon 3 produces an early STOP codon and prevents the expression of luciferase. However, if all three exons are correctly spliced, the luciferase ORF is translated in-frame as a fusion protein with the AGA protein sequence coded by exons 1–3. Thus, the effect of a treatment of cells with substances that enhance splicing can be measured using luciferase activity, measured as emission of visible light.

### 3.3. Xanthine Derivatives Enhance the Correct Splicing of the Mutated AGA mRNA

**Figure 5:**
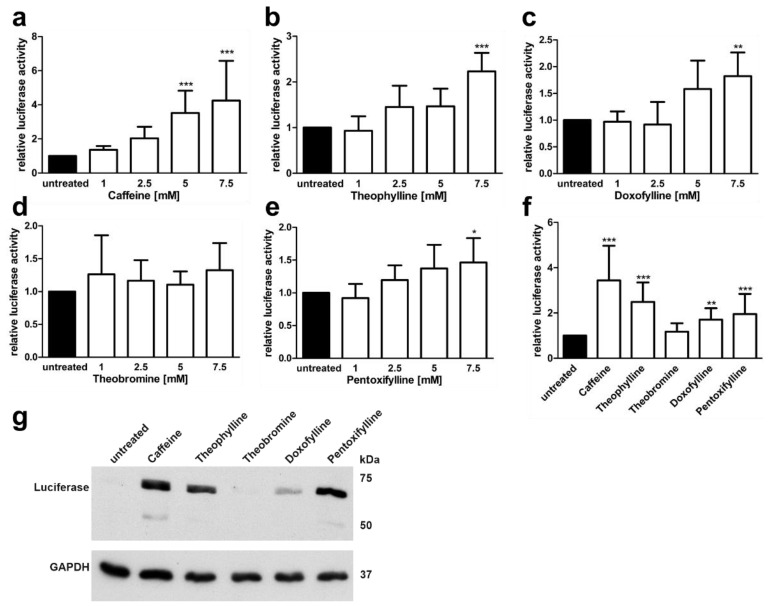
*AGA*-Minigene-luciferase splicing assay in HEK293T cells: Effect of methylxanthines. (**a**–**e**) HEK293T cells were transfected with the *AGA*-minigene construct and treated with increasing amounts of the substances for 24 h. (**f**) The cells were treated with 7.5 mM of each substance. Relative luciferase activity (firefly/renilla luciferase) of the untreated samples was set as 1. Bars represent the mean ± SD of at least five independent experiments. Statistical analysis by one-way ANOVA against the untreated samples. Values of *p* < 0.05 were considered significant (*) while values of *p* < 0.01 were considered very significant (**) and *p* < 0.001 extremely significant (***). (**g**) Protein lysates from (**f**) were analyzed by SDS-PAGE and Western blot. Luciferase expression corresponds to the luciferase activity seen in (**f**).

Natural substances that are derived from xanthine, such as caffeine and theophylline, have been shown to stabilisz mRNAs and to exhibit splicing enhancer activity [25]. Using the *AGA* minigene assay, we tested the capability of five xanthine derivatives to dose dependently enhance the splicing of the *AGA* minigene containing the splice variant (Figure 5). Four different concentrations of the substances (1 mM to 7.5 mM) were tested in our *AGA* knockout HEK293T cell line [23] transfected with the *AGA* minigene construct. The cells were treated with the substances for 24 h, lysed, and the firefly and renilla luciferase activities were measured. Caffeine (1,3,7-trimethylxanthine) treatment resulted in a dose dependent, significant (at 5 mM and 7.5 mM) increase in the luciferase activity as compared to untreated cells (Figure 5a). Similarly, 7.5 mM theophylline (1,3-dimethylxanthine), doxofylline (7-(1,3-dioxolan-2-ylmethyl)-1,3-dimethylpurine-2,6-dione) and pentoxifylline (3,7-dimethyl-1-(5-oxohexyl) xanthine) also significantly increased luciferase activity (Figure 5b–d), whereas theobromine (3,7-dimethylxanthine) resulted in non-significant changes (Figure 5e). Figure 5f shows a direct comparison of the substances at 7.5 mM, with caffeine showing the highest increase, but also the largest variation. Figure 5g shows a Western Blot for the AGA-luciferase fusion protein expression in cells treated with the 7.5 mM compounds.

**Figure 6:**
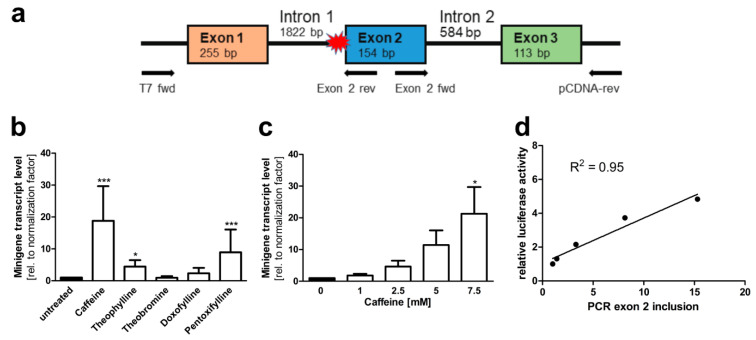
*AGA* minigene qRT-PCR splicing assay in HEK293T cells. (**a**) The *AGA* minigene construct without firefly luciferase, the qRT-PCR primers for detecting exon 2 inclusion are shown with arrows. (**b**,**c**) HEK293T cells were transfected with the minigene construct and treated with (**b**) 7.5 mM of the substances or (**c**) increasing doses of caffeine for 24 h. Total RNA was isolated, reversely transcribed and analyzed by qRT-PCR. Bars represent the mean ± SD of three independent experiments. Statistical analysis by one-way ANOVA against the untreated samples. Values of *p* < 0.05 were considered significant (*) while values of *p* < 0.001 were considered extremely significant (***). (**d**) The caffeine effect from luciferase assay (shown in Figure 5a) was correlated with the caffeine effect in the qRT-PCR assay shown in (**c**).

To verify that the treatment of the cells resulted in inclusion of exons 2 and 3 at the mRNA level, a second minigene construct without the luciferase coding sequence was cloned in the pCDNA3 vector (Figure 6a). *AGA* knockout HEK293T cells were transfected with this construct and treated with the above substances. RNA was isolated from the cells, reverse transcribed to cDNA, and a qRT-PCR with a fwd primer residing in exon 2 and a reverse primer in the plasmid vector (3′-untranslated region of the resulting RNA) was performed. Thus, amplification of a PCR product of 271 bp is only observed when both exons 2 and 3 are present in the mRNA. Examples of the quality control data for the qPCR primers used for the minigene constructs are shown in Supplementary Figures S2 and S3. Figure 6b shows that 7.5 mM caffeine resulted in a significant, almost 20-fold increase in the correctly spliced product. Pentoxifylline and theophylline also significantly increased the correct splicing up to about 5 to 10-fold, whereas doxofylline and theobromine did not produce significant changes in the splicing. This assay also showed a good dose dependency (Figure 6c), and there was a good correlation (R^2^ = 0.95) between the mRNA and luciferase-based minigene assays (Figure 6d). To verify not only the presence of exon 2, but also its correct in-frame fusion to exon 1, a second type of PCR was performed with a T7 fwd vector primer (recognizing the 5′UTR of the minigene transcript) and a reverse primer located in exon 2. Supplementary Figure S4 shows the sequencing data for the dominant amplified product, demonstrating the correct fusion of exons 1 and 2.

**Figure 7:**
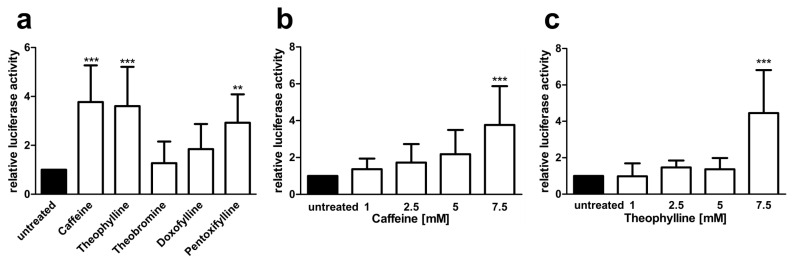
*AGA*-luciferase minigene splicing assays in patient fibroblasts (**a**–**c**) Patient fibroblasts were transfected with the *AGA*-luciferase minigene construct and treated with (**a**) 7.5 mM of substances or (**b**) increasing amounts of caffeine or (**c**) theophylline for 24 h. Relative luciferase activity of the untreated samples was set as 1. Bars represent the mean ± SD of at least five independent experiments, statistics with one-way ANOVA against the untreated samples. Values of *p* < 0.01 were considered very significant (**) and *p* < 0.001 extremely significant (***).

Since the genetic background can have a substantial effect on splicing, we also tested the minigene splicing assays in the AGU patient fibroblasts that were transfected with the *AGA*-luciferase minigene constructs and then treated with 7.5 mM xanthine derivatives as above (Figure 7). As with the HEK293T cells, caffeine and theophylline showed a significant, dose dependent increase in the luciferase readout (Figure 7a–c), and pentoxifylline also significantly increased the luciferase activity at 7.5 mM, whereas theobromine and doxofylline failed to do so (Figure 7a).

**Figure 8:**
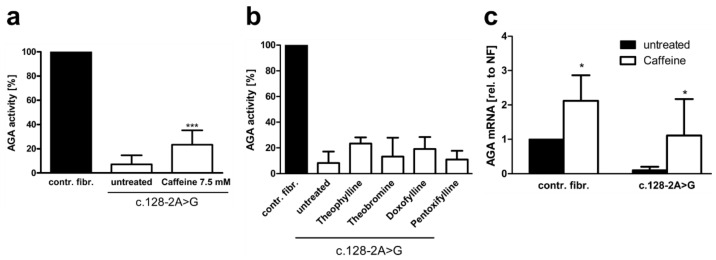
Effect of xanthine derivatives on the AGA activity and *AGA* mRNA level in AGU fibroblasts. Patient fibroblasts were treated for 48 h with 7.5 mM caffeine (**a**) or other methylxanthines (**b**), and the AGA activity was measured; *n* = 4, graphs show the mean ± SD. Statistical analysis by one-way ANOVA. (**c**) Patient and control fibroblasts were treated with 7.5 mM caffeine for 24 h. *AGA* transcript level was measured with qPCR using primers for exons 2 and 3, *n* = 6, two-way ANOVA. Values of *p* < 0.05 were considered significant (*)and *p* < 0.001 extremely significant (***).

The above data pointed to caffeine as the most efficient splicing enhancer for the patient variant studied here. We thus tested the effect of the above substances on splicing and AGA enzyme activity in patient fibroblasts (Figure 8). Caffeine treatment resulted in significant increase of the AGA enzyme activity up to 23.4% of the control fibroblast activity (Figure 8a), whereas all other substances failed to increase the AGA activity to a significant degree (Figure 8b). Consistent with the enzyme activity data, the endogenous *AGA* mRNA splicing products that contain exons 2 and 3 were significantly increased to a level that corresponds to the untreated control fibroblasts, albeit with a high SD (Figure 8c). Interestingly, caffeine treatment also resulted in an increase in the *AGA* mRNA in control fibroblasts, which is consistent with the general mRNA stabilizing effect of caffeine.

### 3.4. Plant-Derived Bioactive Compounds Enhance the Splicing of the AGA mRNA

While caffeine treatment looked quite promising according to our data above, we tested further natural substances that may enhance splicing, since caffeine may be a suboptimal substance for the treatment of pediatric patients. Luteolin (3’,4’,5,7-tetrahydroxyflavone) is a plant-derived flavonoid that has been shown to enhance splicing at some non-canonical, mutated splice-acceptor sites in a fibronectin-1 mini-gene assay, but the study did not extend the findings to an effect on full-length protein function [26]. Kinetin (6-furfuryl-adenine) is a member of the cytokinin plant hormone family, and it has been shown to enhance the splicing in the *IKBKAP* gene carrying a splice-donor site mutation [27,28].

**Figure 9:**
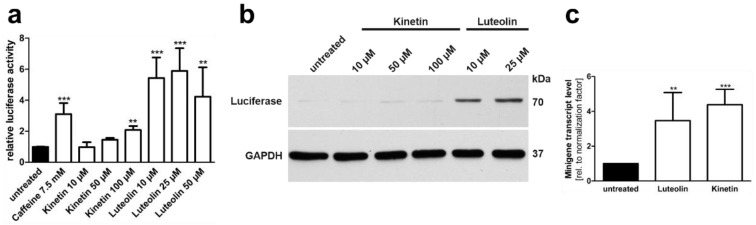
Minigene splicing assays in HEK293T cells: Effect of luteolin and kinetin. (**a**) *AGA* knockout HEK293T cells were transfected with the minigene-luciferase construct and treated with increasing amounts of substances for 24 h. Relative luciferase activity of the untreated samples was set as 1. Bars represent the mean ± SD of at least three independent experiments, one-way ANOVA against the untreated samples. (**b**) Protein lysates from (**a**) were analysed by SDS-PAGE and Western blot. Luciferase expression corresponds to the luciferase activity shown in (**a**). (**c**) *AGA* knockout HEK293T cells were transfected with the *AGA* minigene construct without luciferase and treated with 25 µM luteolin or 50 µM kinetin for 24 h. Total RNA was isolated, reversely transcribed and analyzed by qRT-PCR. Bars represent the mean ± SD of six independent experiments, one-way ANOVA against the untreated samples. Values of *p* < 0.01 were considered very significant (**) and *p* < 0.001 extremely significant (***).

We tested the effect of luteolin and kinetin on the splicing of the *AGA* mRNA with the c.128-2A>G variant. *AGA* knockout HEK 293T cells were transfected with the *AGA*-luciferase minigene, treated with 10–100 µM kinetin or 10–50 µM luteolin, and the luciferase activity was measured in cell lysates (Figure 9a). Treatment with 100 µM kinetin resulted in a significant (about two-fold) increase in luciferase activity, whereas the lower concentrations showed only minor effects. In contrast, all luteolin concentrations resulted in a significant increase in luciferase activity, with 25 µM luteolin being the most efficient concentration (about 5 to 6 fold increase). Consistently, a strong luciferase expression in Western blot was only observed in luteolin treated lysates (Figure 9b). In contrast, the qPCR-based minigene assay showed that both luteolin and kinetin increased the amount of *AGA* mRNA products containing exon 2 (Figure 9c).

**Figure 10:**
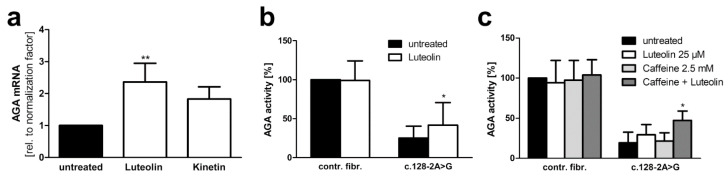
Luteolin treatment increases *AGA* mRNA amount and AGA activity in c.128-2A>G AGU patient fibroblasts. (**a**) Quantitative real-time PCR of *AGA* mRNA with patient fibroblasts with c.128-2A>G variant after treatment with luteolin (25 µM) or kinetin (50 µM) for 24 h. *AGA* was detected with the primers specific for exons 2 and 3. Bars represent the mean ± SD of three independent experiments, one-way ANOVA against the untreated sample. (**b**) Treatment of fibroblasts with luteolin (25 µM) for 48 h and measurement of AGA activity with AADG as substrate, 13 independent experiments, two-way ANOVA against the untreated sample. (**c**) Control and patient fibroblasts were treated with suboptimal amounts of caffeine (2.5 mM) to prevent mRNA degradation, either alone or in combination with luteolin (25 µM) for 48 h. AGA activity was measured as in (**b**), three independent experiments, one-way ANOVA against the untreated sample. Values of *p* < 0.05 were considered significant (*) while values of *p* < 0.01 were considered very significant (**).

When the patient fibroblasts were treated with luteolin or kinetin, the endogenous *AGA* mRNA with exon 2 was significantly increased by luteolin (about 2.5 fold), whereas kinetin treatment showed no significant increase (Figure 10a). Due to these encouraging data with luteolin, the AGA activity was measured in patient fibroblasts treated with 25 µM luteolin (Figure 10b). Since luteolin acts as a quencher in our standard fluorescence-based AGA activity measurement ([29] and our own observations), we used a colorimetric activity assay [30] that is less sensitive but not subject to quenching by luteolin. Indeed, luteolin treatment resulted in a significant increase in the AGA activity in the patient fibroblasts but not in the control fibroblasts (Figure 10b).

The mechanisms of action of caffeine and luteolin on splicing are not identical, and a combination therapy with both substances might facilitate the use of lower concentrations of especially caffeine in the patient. Thus, control and patient fibroblasts were treated with 25 µM luteolin, 2.5 mM caffeine, or a combination thereof. Indeed, combination treatment with both substances resulted in a substantially higher AGA activity than either luteolin or caffeine alone, whereas no changes were observed in the control fibroblasts (Figure 10c). The correct splicing of exon 1–exon 2 junction upon luteolin treatment was verified by sequencing (Supplementary Figure S5).

### 3.5. Xanthine Derivatives and Luteolin Correct the Splicing of a Common cLINCL/TPP1 Variant

**Figure 11:**
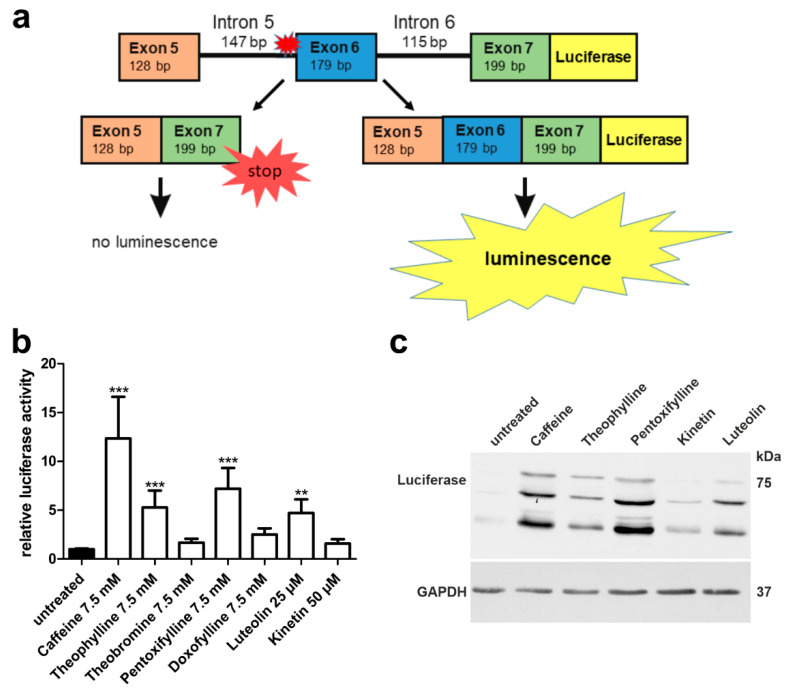
*TPP1* minigene luciferase splicing assay in HEK293T cells: Effect of methylxanthines, luteolin, and kinetin. (**a**) Schematic drawing of the *TPP1* minigene construct containing the variant c.509-1G<C, with C-terminal firefly luciferase. (**b**) HEK293T cells were transfected with the *TPP1* luciferase minigene construct containing the variant c.509-1G<C and treated with the indicated substances for 24 h. Relative luciferase activity of the untreated samples was set as 1. Bars represent the mean ± SD of at least five independent experiments, one-way ANOVA against the untreated samples. Values of *p* < 0.01 were considered very significant (**) and *p* < 0.001 extremely significant (***). (**c**) Protein lysates from (**b**) were analyzed by SDS-PAGE and Western blot with an anti-luciferase antibody. Luciferase expression was observed in the samples showing significant luciferase activity in (**b**).

Splice-site mutations are relatively rare in AGU, whereas in some other lysosomal disorders, they represent the most frequent disease-causing variants found in patients. Classic late neuronal ceroid lipofuscinosis (cLINCL), also known as CLN2 disease, is caused by mutations in the *TPP1* gene that encodes the soluble lysosomal enzyme tripeptidyl peptidase 1 (TPP1). One of the most common disease-causing variants in cLINCL is a splice-acceptor site mutation c.509-1G>C that is present in 27% of the reported patient alleles [31]. In this variant, the splice-acceptor consensus sequence at the 3′ end of intron 5 has been abolished, thus potentially resulting in the exclusion of exon 6 upon splicing of the *TPP1* pre-RNA (Figure 11a). Loss of exon 6 produces a frameshift and early termination of translation after 99 amino acids encoded by exon 7.

In view of the encouraging data obtained with our AGU splice-site variant, we constructed a *TPP1* luciferase minigene that contains exons 5–7 together with the introns 5 and 6 (Figure 11a) and the firefly luciferase coding region. Different from our AGA minigene, we did not include *TPP1* exon 1 due to size restrictions, but an ATG start codon was added in front of exon 5. The splice-site variant -1G>C was introduced at the end of intron 5. Upon exclusion of exon 6, which is the predicted alteration due to the splice-site mutation, no luciferase activity is observed due to the frameshift and premature stop codon. Other altered splicing products are possible, but they would not result in a luciferase signal. If the splicing can be corrected, and exon 6 is included, luciferase activity can be measured (Figure 11a).

Significant increase of luciferase signal was detected with 7.5 mM caffeine, theophylline, pentoxifylline, and with 25 µM luteolin (Figure 11b). Luciferase expression was verified by Western blot (Figure 11c) and was in accordance with the activity readouts from Figure 11b.

**Figure 12:**
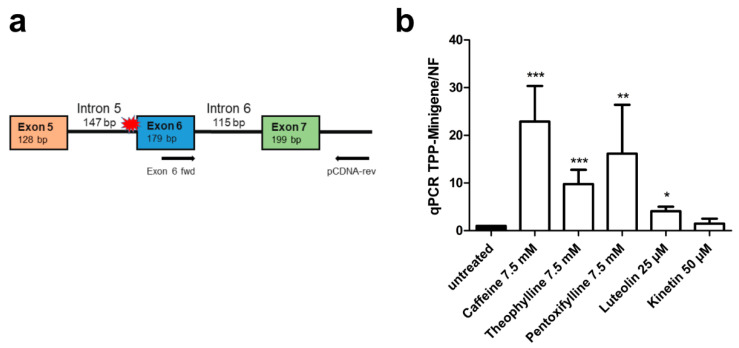
*TPP1* minigene qRT-PCR splicing assays in HEK293T cells. (**a**) Schematic of the *TPP1* minigene construct containing the variant c.509-1G<C without firefly luciferase. The qRT-PCR primers are shown with arrows. (**b**) HEK293T cells were transfected with the *TPP1* minigene construct and treated with caffeine (7.5 mM), theophylline (7.5 mM), pentoxifylline (7.5 mM), luteolin (25 µM) or kinetin (50 µM) for 24 h. Total RNA was isolated, reversely transcribed and analyzed by qRT-PCR. Bars represent the mean ± SD of three independent experiments, one-way ANOVA against the untreated samples. Values of *p* < 0.05 were considered significant (*), while values of *p* < 0.01 were considered very significant (**) and *p* < 0.001 extremely significant (***).

Analogously to our approach with the *AGA* splicing assay, we also constructed a *TPP1* minigene without luciferase to demonstrate the presence of exon 6 and correction of splicing upon treatment with caffeine, theophylline, pentoxifylline, luteolin and kinetin by qRT-PCR (Figure 12a). In accordance with the luciferase assay, significantly increased inclusion of exon 6 was observed after caffeine, theophylline, pentoxifylline, and luteolin treatment, whereas kinetin did not increase exon 6 inclusion (Figure 12b). Examples of the quality control data for the qPCR experiments are provided in Supplementary Figure S6, and the sequence of the amplicon, showing the correct joining of exons 6 and 7, is shown in Supplementary Figure S7.

### 3.6. Role of Splicing Factors in the Splicing of the Mutant AGA Gene

**Figure 13:**
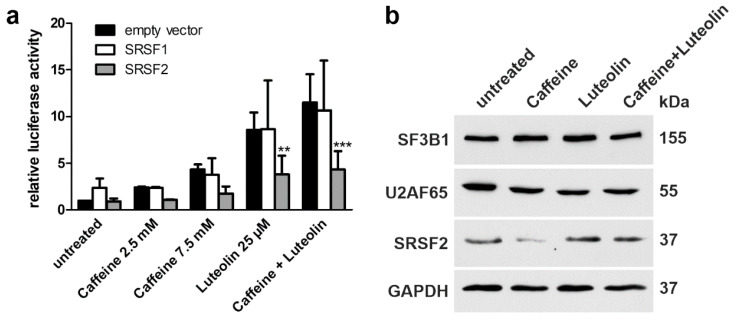
Expression of SRSF upon luteolin and caffeine treatment. (**a**) HEK293T cells were cotransfected with equal amounts of the *AGA*-luciferase minigene construct and the expression constructs for SRSF1, SRSF2, or empty pEXPR-IBA103. Cells were treated with the indicated amounts of the substances for 24 h. Relative luciferase activity of the untreated samples cotransfected with the empty vector was set as 1. Bars represent the mean ± SD of at least three independent experiments, two-way ANOVA against the samples cotransfected with the empty vector. Values of *p* < 0.01 were considered very significant (**) and *p* < 0.001 extremely significant (***). (**b**) Expression level of splice factors in patient fibroblasts. Cells were treated with caffeine (7.5 mM), luteolin (25 µM) or a combination of 2.5 mM caffeine and 25 µM luteolin for 48 h. Cell lysates were used for Western blotting. GAPDH served as a loading control. Results are representative for three independent experiments.

The members of the serine and arginine rich splicing factor (SRSF) family are important components of the splicing machinery. Splicing factors of the SRSF protein family, such as SRSF2 (also called SC35), bind to exonic splicing enhancers (ESE) and are able to modulate splicing by regulation of the spliceosome assembly (for review, see [32,33]). Therefore, we tested if overexpression of Strep-tagged SRSF1 and SRSF2 could improve the splicing of the *AGA*-luciferase minigene (Figure 13a). HEK293T cells were transfected with the respective expression plasmids, treated with caffeine (2.5 mM or 7.5 mM), luteolin (25 µM), or a combination thereof (Figure 13a, verification of the expression of SRSF proteins in Supplementary Figure S8). While the treatment with the substances resulted in an increased luciferase activity, indicating enhanced correct splicing, SRSF overexpression did not enhance the splicing further. Interestingly, overexpression of SRSF2 caused a lower luciferase activity in treated cells, indicating an inhibitory effect (Figure 13a). Furthermore, overexpression of SRSF1 appeared to enhance cryptic splicing of intron 1 upon luteolin treatment, since 23 nucleotides from the 3′ end of intron 1 were frequently found in the sequenced minigene splicing product (Supplementary Figure S8).

Caffeine has been shown to increase the expression of SRSF2 in cancer cells and to alter the splicing of cancer-related proteins [34,35,36]. Therefore, we tested if treatment with caffeine and/or luteolin would increase the expression of splicing factors in patient fibroblasts. In addition to SRSF2, two splicing factors that have been suggested to directly bind luteolin and apigenin were studied [37]. SF3B1 (splicing factor 3b subunit 1) and U2AF65 (splicing factor U2AF 65 kDa subunit) are components of the SF3b complex that binds the pre-mRNA 5′ of the intron branching site, together with SF3a. Since double transfection experiments in patient fibroblasts are not feasible due to the low transfection efficiency, we analyzed the expression of endogenous proteins (Figure 13b). Treatment of patient fibroblasts did not result in any consistent changes in the expression of SRSF2, SF3B1, or U2AF65, although in some experiments, the expression of SRSF2 appeared to be reduced by caffeine (Figure 13b).

## 4. Discussion

In this manuscript, we have dissected the effect of several compounds on the splicing of pre-mRNAs that carry splice-acceptor site mutations. The tested compounds have been shown to have a beneficial effect on either mRNA stability and/or pre-mRNA splicing. We here show that some derivatives of xanthine, especially caffeine, and the plant-derived flavonoid luteolin are able to significantly correct the splicing of *AGA* pre-mRNA mutated at position -2 preceding exon 2 in a minigene splicing assay. In addition, caffeine and luteolin significantly increased the amount of correctly spliced *AGA* mRNA and AGA enzyme activity in patient fibroblasts homozygous for the said gene defect. Importantly, our data also show that the splicing of the *TPP1* gene deficient in another lysosomal storage disorder, cLINCL, can be improved in a minigene assay using the same substances. Thus, our findings suggest that these substances may be relevant for the therapy of diseases that result from splice-acceptor site mutations.

Regulation of mRNA splicing has attracted substantial attention in recent years due to its relevance for numerous human diseases, including cancer and many genetic disorders (for a recent review, please see [38]). Alternative splicing of genes is a very common process, and a vast majority of human pre-mRNAs have been suggested to be subjected to alternative splicing [39]. Changes in alternative splicing due to genetic mutations in the splice-sites are the underlying cause in about 15% of genetic diseases [9]. In some diseases such as cLINCL, splice-site mutations may even be among the most common disease-causing gene variants. Therefore, therapy approaches that aim at correcting the splicing defect may in some cases be beneficial for a large fraction of patients. For example, the cLINCL variant analyzed in this study is present in 27% of patient alleles [31], making splicing therapy an attractive option in this disease.

Our AGU patient is homozygous for a novel gene variant that has so far not been reported in further AGU patients. However, the parents originate from the same geographical region in India, but they were not found to be related with each other. Therefore, although AGU has been assumed to be extremely rare outside of Finland, this may imply that the gene variant found in our patient may be more common in this specific region in India, suggesting a founder effect, similarly to the AGU_Fin-major_ variant in Finland. Therefore, it might be possible to identify further AGU patients in this region in India.

Our data show that caffeine and luteolin, or a combination thereof, would be the most promising therapy option for the AGU splice variant described in our study. In addition, theophylline and pentoxifylline also produced significant results in the *AGA* minigene assay. The effect of these substances on splicing and mRNA stability has been demonstrated before in numerous studies. Below, we briefly summarize the current literature to these substances as splice modulators and discuss their potential use as therapeutic agents for splicing defects.

Among many other effects (e.g., cell cycle regulation, neuroactive effects, adenosine receptor modulation, phosphodiesterase inhibition), caffeine has been shown to interfere with gene expression by either changing the expression of splicing enhancers and/or by stabilizing mRNAs containing nonsense-codons (for a review, see [40]). Caffeine induces the expression of SRSF2 and downregulates the expression of SRSF3, and it was shown to regulate alternative splicing of the tumor suppressors p53 and Kruppel like factor 6 (KLF6) in cancer cells [34,35,36]. However, we did not observe any significant and consistent changes in the expression of the splicing factors SRSF2, SF3B1 and U2AF65 in our patient fibroblasts treated with luteolin and/or caffeine. This may be due to different effects of these substances in primary cells (our study) vs. cancer cells that react with a loss of viability to substances like luteolin [37]. While the exact mechanism how luteolin and caffeine enhance the splicing of mutated splice-sites could not be dissected in our study, merely increasing the expression of splice regulatory proteins such as SRSF1 and SRSF2 seems not to be sufficient. In fact, overexpression of SRSF1 even increased cryptic splicing from an intronic site, resulting in an inclusion of extra nucleotides in the mRNA. Therefore, further studies will be required to dissect the mechanism of action of luteolin and caffeine at the molecular level.

Caffeine can attenuate nonsense-mediated decay (NMD) by inhibiting the kinase SMG1 (nonsense mediated mRNA decay associated, PI3K related kinase), which is responsible for the phosphorylation and activation of the RNA helicase and ATPase UPF1 (Up-frameshift 1), a main component of the classical NMD [41]. In cell culture studies, caffeine has been shown to improve the read-through capacity of Ataluren and aminoglycosides [42,43]. In some epidemiological studies, caffeine exhibited moderate neuroprotective effects in neurodegenerative diseases such as Alzheimer’s and Parkinson’s disease (for a review, see [44]).

Due to its bronchodilatating properties, theophylline has been used for many years to treat airway diseases such as COPD (chronic obstructive pulmonary disease) and asthma, but it has been increasingly replaced by other medications that provide a better therapeutic window and less side effects [45,46]. Similar to caffeine, also theophylline was shown to interfere with the expression of SRSF2, SRSF3 and alternative splicing of p53 and KLF6 [36,47]. Theophylline may also exert its effects on gene expression by means of activation of histone deacetylases (HDACs) [48]. However, due to its pronounced effects on the heart, kidney and lung function as well as gastrointestinal effects [49], theophylline may not be particularly suitable at least for a long-term therapy in pediatric patients, although our data suggested that it may be capable of ameliorating the splicing defects addressed in the present study.

Doxofylline is a synthetic methylxanthine that contains a dioxalane group. It also shows bronchodilatating properties, but has a better tolerability and an improved therapeutic window as compared to theophylline. Unlike other xanthines, doxofylline lacks affinity for adenosine receptors and does not produce stimulant effects, which might improve its suitability as a therapeutic agent (for review, see [45]). However, there are no reports so far on an involvement of doxofylline in splicing regulation. Doxofylline also failed to exert any significant effects on splicing of the *AGA* and *TPP1* minigenes in our study.

While pentoxifylline showed some promise especially in enhancing *TPP1* splicing in our minigene assay, it is currently not clear if it would be suitable for a long-term therapy in pediatric patients, due to its potential side effect profile. Pentoxifylline is a xanthine derivative that exhibits, e.g., anti-fibrotic and anti-inflammatory properties, and it is used to improve blood flow in human diseases due to its vasodilatative effects [50,51]. Similar to caffeine and theophylline, pentoxifylline has been suggested to be able to increase the expression of SRSF2 [36]. It was also suggested that caffeine exerts its effect on SRSF2 expression by downregulating micro-RNAs that normally repress SRSF2 expression. Enhanced SRSF2 translation then affects the splicing and stability of SRSF2 mRNA, further increasing its own expression [36]. However, our data show that the splice-enhancing effects observed in our study are not due to enhanced expression of splicing factors, and further mechanisms are likely to exist.

Luteolin and kinetin are plant-derived substances that have been shown to affect gene splicing albeit only in mini-gene assays, and no effect on functional restoration of the encoded protein, particularly in cellular environment, has been shown. Interestingly, these compounds appear to have distinct potential in enhancing splicing at mutated splice-sites. Kinetin has been suggested to be effective in enhancing the splicing of mutated splice-donor sites [27,28,52,53], whereas luteolin has been shown to improve splicing of some weak splice-acceptor sites in mini-gene assays [37,54]. Consistent with these findings, kinetin did not show a systematic positive effect on the splicing of the *AGA* and *TPP1* variants analyzed in our study.

In the present study, luteolin, especially in combination with a low dose of caffeine, significantly increased correct splicing of *AGA* mRNA and also increased AGA activity in AGU patient cells homozygous for the c.128-2A>G *AGA* gene variant, demonstrating that functional restoration of the protein can be achieved with luteolin. Luteolin is a plant-derived bioactive flavonoid with multiple biological effects in cell culture studies, such as anti-inflammatory, anti-allergic and anti-carcinogenic effects [55,56,57,58]. As a naturally occurring flavonoid, luteolin is a normal ingredient of human diet. High levels are typically found in celery, sweet bell peppers, carrots, broccoli, thyme, rosemary, oregano and parsley [59].

High uptake of luteolin has been shown to be associated with health benefits such as lower risk of acute myocardial infarction and ovarian cancer [60,61]. However, a direct demonstration of the benefits of luteolin in diseases such as cancer would still need to be addressed in clinical studies with defined amounts of luteolin. Luteolin has been tested in clinical studies involving pediatric patients with autism spectrum disease [62,63]. Oral application of capsules containing luteolin and quercetin, another flavonoid, was shown to be effective in reducing aberrant behavior and in increasing adaptive functioning in pediatric patients with autism spectrum disorder, and no major adverse effects, except for temporary irritability, were observed [62]. However, these studies have not directly connected the positive effects of luteolin with altered splicing. On the other hand, luteolin and the related compound apigenin have been shown to enhance splicing at non-canonical, weak splice acceptor sites in a fibronectin-1 minigene assay [26] and to alter the splicing patterns of some genes [37,54]. Luteolin was identified in a screen for molecules that correct splicing at mutated splice acceptor sites, particularly when the acceptor site is -1G->T, but the study did no show whether it had any effect on the fibronectin-1 protein function, and no effect was also shown in an in vivo cell environment on the amount or function of the protein [26]. Therefore, our data here suggest for the first time that luteolin and possibly also other flavonoids may exhibit potential as splice modulators that can be used for therapy of diseases resulting from gene variants that affect splicing.

## 5. Conclusions

Modulation and correction of splicing by small molecules such as flavonoids is a highly interesting therapy option. Some studies have shown that flavonoids and other substances can modulate splicing of a large number of genes and may even induce apoptosis [37,54]. However, most of the studies addressing small molecules as splicing modulators have been performed in cancer cells, and food-derived compounds, including flavonoids, show potential for the treatment of cancers (reviewed in [24]). While apoptosis induction and general impairment of splicing has been observed with apigenin and luteolin in cancer cells, non-tumorigenic cells show much weaker effects on splicing in general and on cell viability [37]. In view of the prior knowledge, we were pleased to observe that in the present study, we did not detect any major effects on cell viability in our primary patient fibroblasts treated with luteolin. Nevertheless, RNA sequencing studies that address the effect of luteolin and other flavonoids on the cellular transcriptome as a whole should be performed in suitable patient cells, such as iPSC-derived neurons, to minimize any potential aberrant effects of flavonoids and to make sure that they are suitable for a long-term treatment of vulnerable pediatric patients.

The present study was carried out using both minigene approaches (for *AGA* and *TPP1*) and patient fibroblasts homozygous for the gene variant in question (for *AGA*). Due to the promising data obtained in our study, and particularly the encouraging results in the patient cells, we believe this treatment could be useful for correcting splicing defects in a variety of genes, and provides a useful treatment option for diseases involving splice acceptor-site mutations in general. It would be interesting to expand these studies to further cell models, such as neuronal cells or even organoids generated from patient-derived induced pluripotent stem cells (iPSC), where, based on our data, it is reasonable to expect a therapeutic effect with these molecules. Unfortunately, such cell models are currently not available for the *AGA* gene defect addressed in our study. In addition, further substances that modulate splicing could be identified in a drug screen. On the other hand, luteolin has been shown to be able to cross the blood–brain barrier [64], and it has been used in pediatric patients [62], suggesting that it is safe and, in view of our study, may be suitable for the treatment of neurodegenerative diseases resulting from intronic splice acceptor site mutations in diseases, such as lysosomal storage disorders.

## 6. Patents

Patent application No. EP 21173645.9 has been filed.

## Figures and Tables

**Table 1:** Primer sequences for cloning of the *AGA* and *TPP1* minigene constructs. All sequences are shown in 5′ to 3′ direction.

Primer Name	Primer Sequence 5′ to 3’
AGA-Minigene fwd HindIII	CTATAAAGCTTAGGGACGCCTGAGCGAACCC
AGA-Minigene rev XhoI	CTATACTCGAGCTGACTCTCCTACTAAAAGTGTGT
TPP-IVS-Minigene fwd BamHI	CTAGGATCCATGCAAGCAGAGCTGCTGCTCCCTG
TPP-IVS-Minigene rev XhoI	CTATACTCGAGCAGGGCTACTGTAGACCCAGGTG
Luciferase fwd XhoI	CTATACTCGAGAAGACGCCAAAAACATAAAGAAAGG
Luciferase rev XbaI	CTATATCTAGATTACACGGCGATCTTTCCGCC

**Table 2:** Sequences of primers used for the quantitative real-time PCR. All sequences are shown in 5′ to 3′ direction.

Primer Name	Primer Sequence 5′ to 3′
B2M-fwd	AGATGAGTATGCCTGCCGTGTG
B2M-rev	TGCGGCATCTTCAAACCTCCA
Rpl13a-fwd	CCTGGAGGAGAAGAGGAAAGAGA
Rpl13a-rev	TTGAGGACCTCTGTGTATTTGTCAA
Ywhaz-fwd	AGGTTGCCGCTGGTGATGAC
Ywhaz-rev	GGCCAGACCCAGTCTGATAGGA
TBP-fwd	GACTATTGGTGTTCTGAATAGGC
TBP-rev	GGAATCCCTATCTTTAGTCCAAT
AGA exon2 fwd	CGGCTCTGTAGGCTTTGGAGGA
AGA exon2 rev	GCCTCCAGATGCTAATGCCCTC
AGA exon3 rev	CCAGTACTTTCCGTGCCACACC
AGA exon 7/8 fwd	ATGGCCGTGTAGGAGACTCACC
AGA exon 8/9 rev	ACAGCTTGGTAGCTTGGCAGGA
TPP1 exon 6/7 fwd	CTGTGCCCAGTTCCTGGAGC
pCDNA3-rev	GGCAACTAGAAGGCACAGTC
T7 fwd	TAATACGACTCACTATAGGG

## Data Availability

The data presented in this study are available on a reasonable request from the corresponding author. The data are not publicly available due to privacy concerns (single patient).

## Supplementary Materials

The following are available online at https://www.mdpi.com/article/10.3390/cells10112813/s1, Figure S1: Sequencing of the patient genomic DNA, Figure S2: Quality control of *AGA* minigene quantitative real-time PCR, Figure S3: Agarose gel for quality control of *AGA* minigene quantitative real-time PCR, Figure S4: Sequencing of the *AGA* minigene product to confirm correct splicing of exon 1/2 junction of AGA, Figure S5: Sequencing of the PCR product from luteolin-treated patient fibroblasts to confirm the correct splicing of exon 1/2 junction of AGA, Figure S6: Quality control of *TPP1* quantitative real-time PCR, Figure S7: Agarose gel for quality control of *TPP1* quantitative real-time PCR and sequencing of PCR products, Figure S8: Overexpression of Strep-tagged SRSF1 and SRSF2.
