# Supplementary material for: Towards Splicing Therapy for Lysosomal Storage Disorders: Methylxanthines and Luteolin Ameliorate Splicing Defects in Aspartylglucosaminuria and Classic Late Infantile Neuronal Ceroid Lipofuscinosis
Source: Cells. 2021 Oct 20;10(11):2813. doi: 10.3390/cells10112813 (PMC8616534; doi:10.3390/cells10112813)
Supplement: Supplementary file 1 [file cells-10-02813-s001.zip › cells-1239964-supplementary.pdf]

Supplementary Figure S1

```
Query  2044  ATATAAACAAGCATTATTGGGATTTGATTAACAGCGTGGAGGGCATTAGCATCTGGAGGC  2103
             |||||||||||||||||||||||||||||||| |||||||||||||||||||||||||||
Sbjct  870   ATATAAACAAGCATTATTGGGATTTGATTAACGGCGTGGAGGGCATTAGCATCTGGAGGC  811

Query  2104  TCTGCCCTGGATGCAGTGGAGAGCGGCTGTGCCATGTGTGAGAGAGAGCAGTGTGACGGC  2163
             ||||||||||||||||||||||||||||||||||||||||||||||||||||||||||||
Sbjct  810   TCTGCCCTGGATGCAGTGGAGAGCGGCTGTGCCATGTGTGAGAGAGAGCAGTGTGACGGC  751

Query  2164  TCTGTAGGCTTTGGAGGAAGTCCTGATGAACTTGGAGAAACCACACTAGATGCCATGATC  2223
             ||||||||||||||||||||||||||||||||||||||||||||||||||||||||||||
Sbjct  750   TCTGTAGGCTTTGGAGGAAGTCCTGATGAACTTGGAGAAACCACACTAGATGCCATGATC  691

Query  2224  ATGGATGGGTAAGAACACCGTTCAGGTCCTATTTATTATGTTGAGTTACATTTAGAGTTG  2283
             |||||||||||||||||||| |||||||||||||||||||||||||||||||||||||||
Sbjct  690   ATGGATGGGTAAGAACACCGGTCAGGTCCTATTTATTATGTTGAGTTACATTTAGAGTTG  631

Query  2284  TGGTCTTCTGAGAGCAAGATGAAAGAATGCATGAGTTTTGACACAGATCAAAACCCCTAG  2343
             ||||||||||||||||||||||||||||||||||||||||||||||||||||||||||||
Sbjct  630   TGGTCTTCTGAGAGCAAGATGAAAGAATGCATGAGTTTTGACACAGATCAAAACCCCTAG  571

Query  2344  CTTATTATACTTGACCTTTCTAAACACAGTTTCCCTCTCAACTGaaaaaaaaaaaTCAGA  2403
             ||||||||||||||||||||||||||||||||||||||||||||||||||||||||||||
Sbjct  570   CTTATTATACTTGACCTTTCTAAACACAGTTTCCCTCTCAACTGAAAAAAAAAAATCAGA  511

Query  2404  GTAATAGAAATATCACATGGAGAGATAGGTAGTCCCTTAGAACTGGGAGAATGCCAAGCT  2463
             ||||||||||||||||||||||||||||||||||||||||||||||||||||||||||||
Sbjct  510   GTAATAGAAATATCACATGGAGAGATAGGTAGTCCCTTAGAACTGGGAGAATGCCAAGCT  451

Query  2464  AAAAAACTATGTCTCTGACCTTATAAACAATCATTTATCTGCTGTCCACAAATGCTGTGG  2523
             ||||||||||||||||||||||||||||||||||||||||||||||||||||||||||||
Sbjct  450   AAAAAACTATGTCTCTGACCTTATAAACAATCATTTATCTGCTGTCCACAAATGCTGTGG  391

Query  2524  TTTGCCTGACCTGAAGCCTTCAGGCCCCTTTCAAAAGCCATATTTAAAATTAGCTTGGAG  2583
             ||||||||||||||||||||||||||||||||||||||||||||||||||||||||||||
Sbjct  390   TTTGCCTGACCTGAAGCCTTCAGGCCCCTTTCAAAAGCCATATTTAAAATTAGCTTGGAG  331

Query  2584  AAACTGAGAATTTTAGTGCTGTTGTGGTACATGGCAAGTTGATTTTCTAttttttttCCT  2643
             |||||||||||||||||||||||||||||||||||||||||||||||||||||||| |||
Sbjct  330   AAACTGAGAATTTTAGTGCTGTTGTGGTACATGGCAAGTTGATTTTCTATTTTTTT-CCT  272

Query  2644  TAATTTTGTCTGATTGTGGGAATCTATATGAACTTCGTGGATGTGCTGTCACTTCTGTGT  2703
             ||||||||||||||||||||||||||||||||||||||||||||||||||||||||||||
Sbjct  271   TAATTTTGTCTGATTGTGGGAATCTATATGAACTTCGTGGATGTGCTGTCACTTCTGTGT  212

Query  2704  TAAGATGGTGGAGTTTAACCGCAAAACACTATTGATTGGAGAAAAGCAATGTTTTTAATC  2763
             ||||||||||||||||||||||||||||||||||||||||||||||||||||||||||||
Sbjct  211   TAAGATGGTGGAGTTTAACCGCAAAACACTATTGATTGGAGAAAAGCAATGTTTTTAATC  152
```

Figure S1. Sequencing of the patient genomic DNA. The region of the *AGA* gene spanning exons 1-3 and introns 1-2, including the mutation c.128-2A>G in intron 1, was PCR-amplified from the AGU patient genomic DNA and cloned into pcDNA3 vector. Plasmid DNA was sequenced to confirm the presence of the mutation. The alignment against the genomic reference sequence of AGA (NG_011845.2, upper row) was performed with the sequence obtained using a reverse primer (lower row). The mutation site is indicated with a red frame.

Supplementary Figure S2

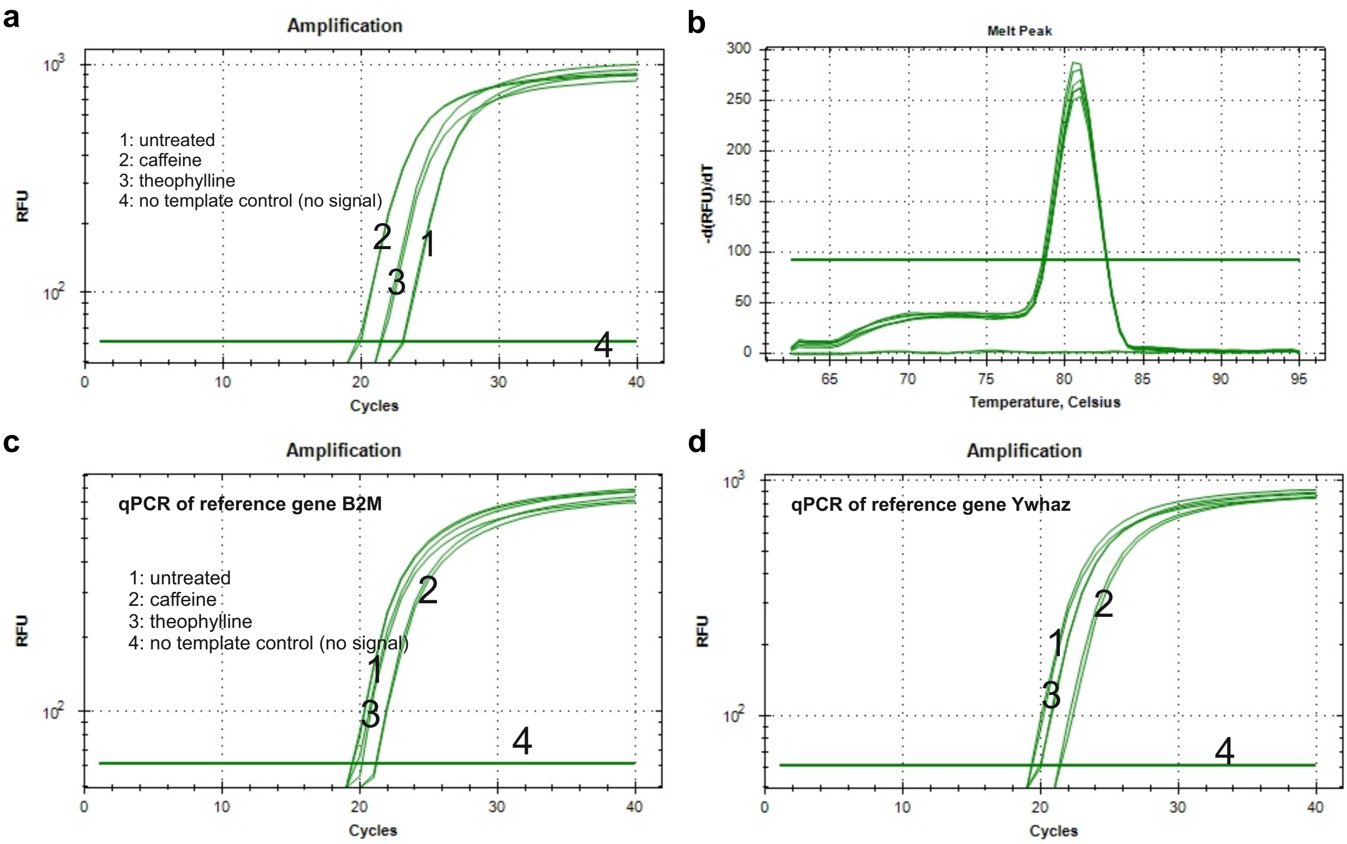

Figure S2. Quality control of *AGA* minigene quantitative real-time PCR. HEK293T cells were transfected with the *AGA* minigene construct and treated with 7.5 mM substances, as indicated. Total RNA was isolated, reversely transcribed and analysed by qPCR. To test for correct splicing, i.e. inclusion of exon 2, qPCR was performed with a forward primer located in exon 2, in combination with the vector primer pcDNA3-reverse. (a) The effects of caffeine and theophylline on the amount of products containing AGA exon 2 can be calculated using the -ΔΔCt method. (b) The melting curve of PCR products from (a) shows only one peak. No signal was detected in the negative control (Number 4, water instead of cDNA). (c, d) For normalization of the qPCR results, the same cDNA samples were used for amplification of reference genes *B2M* and *Ywhaz*.

Supplementary Figure S3

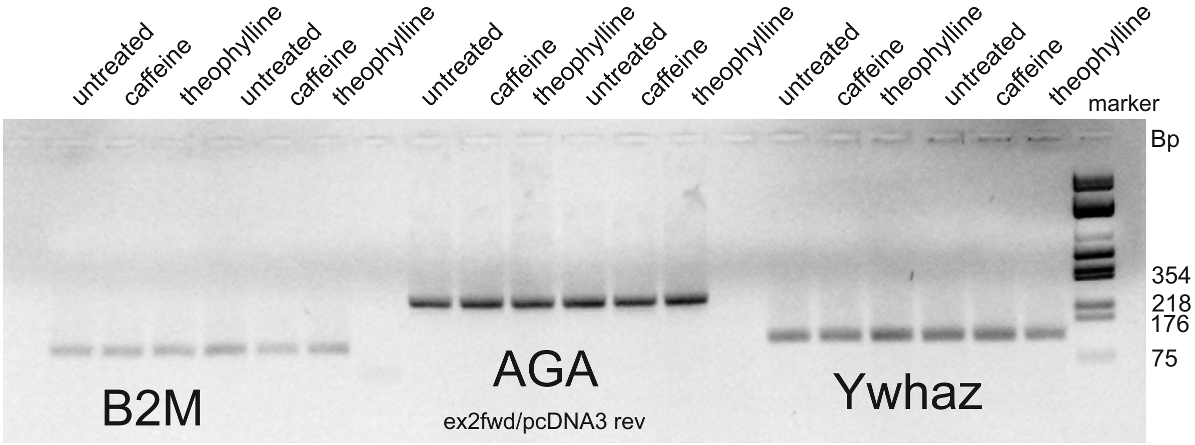

Figure S3. Agarose gel for quality control of *AGA* minigene quantitative real-time PCR. After completion of 40 cycles of quantitative real-time PCR, samples shown in Suppl. Figure S2 were separated on an agarose gel. All samples exhibited only a single band of the expected size. No unspecific products or primer dimers were detected. In addition, DNA bands from the agarose gel were sequenced to confirm that exons 2 and 3 are correctly fused to each other. Note that after 40 cycles of amplification (end-point), the intensity differences between the samples are not visible any more.

Supplementary Figure S4

```
Query  63   ATGGCGCGGAAGTCGAACTTGCCTGTGCTTCTCGTGCCGTTTCTGCTCTGCCAGGCCCTA  122
            ||||||||||||||||||||||||||||||||||||||||||||||||||||||||||||
Sbjct  1    ATGGCGCGGAAGTCGAACTTGCCTGTGCTTCTCGTGCCGTTTCTGCTCTGCCAGGCCCTA  60

Query  123  GTGCGCTGCTCCAGCCCTCTGCCCCTGGTCGTCAACACTTGGCCCTTTAAGAATGCAACC  182
            ||||||||||||||||||||||||||||||||||||||||||||||||||||||||||||
Sbjct  61   GTGCGCTGCTCCAGCCCTCTGCCCCTGGTCGTCAACACTTGGCCCTTTAAGAATGCAACC  120

Query  183  GAAGCAGCGTGGAGGGCATTAGCATCTGGAGGC  215
            ||||||||||||||||||||| |||||||||||
Sbjct  121  GAAGCAGCGTGGAGGGCATTANCATCTGGAGGC  153
```

Figure S4. Sequencing of the *AGA* minigene product to confirm correct splicing of exon 1/2 junction. To confirm the correct fusion of exons 1 and 2, total RNA was isolated from cells transfected with the minigene construct and subsequently treated with 7.5 mM caffeine. The cDNA was PCR amplified with a T7 forward vector primer and a reverse primer specific for *AGA* exon 2. The resulting dominant DNA band was sequenced with the forward primer. The alignment shows an example for the sequenced PCR product (lower row) with the *AGA* reference sequence (NM_000027.4, upper row). Exon 1 is shown in yellow, exon 2 in green. Exons 1 and 2 are correctly fused to each other, demonstrating the correct splicing upon caffeine treatment. The only mismatch towards the end of the sequencing is due to the poor quality of the sequence at this position.

Supplementary Figure S5

```
Query  125   GCGCTGCTCCAGCCCTCTGCCCCTGGTCGTCAACACTTGGCCCTTTAAGAATGCAACCGA  184
             ||||||||||||||||||||||||||||||||||||||||||||||||||||||||||||
Sbjct  1     GCGCTGCTCCAGCCCTCTGCCCCTGGTCGTCAACACTTGGCCCTTTAAGAATGCAACCGA  60

Query  185   AGCAGCGTGGAGGGCATTAGCATCTGGAGGC  215
             |||||||||||||||||||||||||||||||
Sbjct  61    AGCAGCGTGGAGGGCATTAGCATCTGGAGGC  91
```

Figure S5. Sequencing of the PCR product from luteolin-treated patient fibroblasts to confirm the correct splicing of exon 1/2 junction of *AGA*. The junctional region of exons 1 and 2 of the *AGA* cDNA from luteolin-treated AGU patient fibroblasts was amplified and sequenced with the forward primer (for experimental details, see Figure S4). The alignment depicts the sequenced PCR product (lower row) aligned with the *AGA* reference sequence (NM_000027.4, upper row). Exon 1 is shown in yellow, exon 2 in green. Exons 1 and 2 are correctly fused to each other, demonstrating the correct splicing, despite the splice-site mutation in the intron junction preceding exon 2.

Supplementary Figure S6

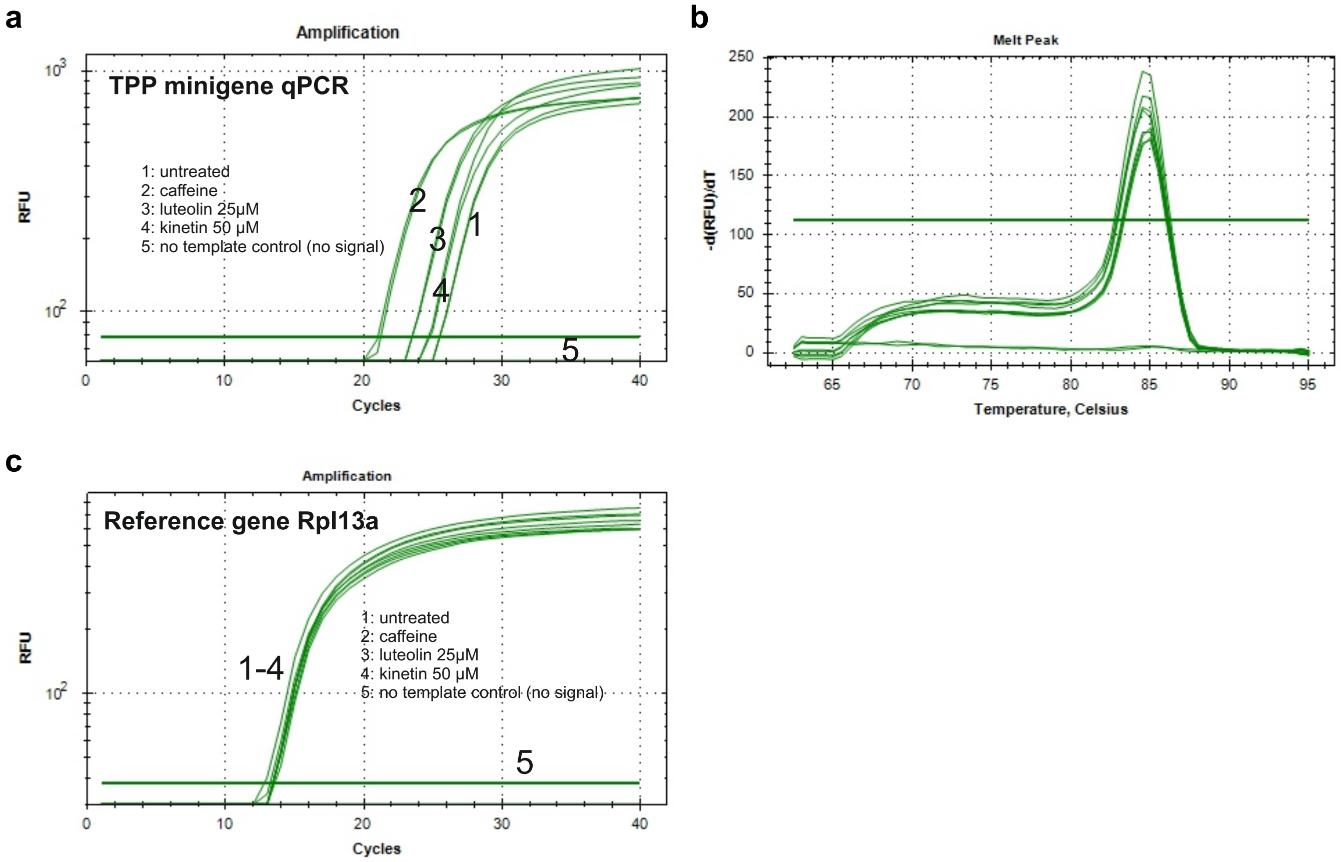

Figure S6. Quality control of *TPP1* quantitative real-time PCR. HEK293T cells were transfected with the *TPP1* minigene construct and treated with the indicated substances. Total RNA was isolated, reversely transcribed and analysed by qPCR. To test for correct splicing, i.e. inclusion of exon 6, qPCR was performed with a forward primer located in exon 6, in combination with the vector primer pcDNA3-reverse. (a) The effects of caffeine and luteolin on the amount of a product containing *TPP1* exon 6 can be calculated using the -ΔΔCt method. (b) The melting curve of PCR products from (a) shows only one peak. No signal was detected in the negative control (Number 4, water instead of cDNA). (c) For normalization of the qPCR results, the same cDNA samples were used for amplification of reference genes. Curves show Ct-values for one of the reference genes used (*Rpl13a*).

Supplementary Figure S7

a

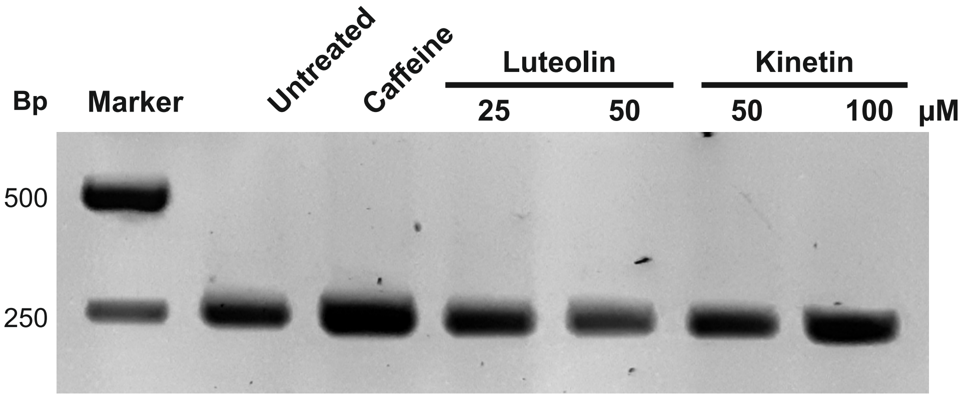

b

```
Query  680   ACCAGCAATAACAGCCAAGCCTGTGCCCAGTTCCTGGAGCAGTATTTCCATGACTCAGAC  739
             ||||||||||||||||||||||||||||||||||||||||||||||||||||||||||||
Sbjct  1     ACCAGCAATAACAGCCAAGCCTGTGCCCAGTTCCTGGAGCAGTATTTCCATGACTCAGAC  60

Query  740   CTGGCTCAGTTCATGCGCCTCTTCGGTGGCAACTTTGCACATCAGGCATCAGTAGCCCGT  799
             ||||||||||||||||||||||| ||||||||||||||||||||||||||||||||||||
Sbjct  61    CTGGCTCAGTTCATGCGCCTCTTNGGTGGCAACTTTGCACATCAGGCATCAGTAGCCCGT  120

Query  800   GTGGTTGGACAACAGGGCCGGGGCCGGGCCGGGATTGAGGCCAGTCTAGATGTGCAGTAC  859
             ||||||||||||||||||||||||||||||||||||||||||||||||||||||||||||
Sbjct  121   GTGGTTGGACAACAGGGCCGGGGCCGGGCCGGGATTGAGGCCAGTCTAGATGTGCAGTAC  180

Query  860   CTGATGAGTGCTGGTGCCAACATCTCCACCTGGGTCTACAGTAGCCCTGGCCG-GCATG  917
             |||||||||||||||||||||||||||||||||||||||||||||||||  || |||||
Sbjct  181   CTGATGAGTGCTGGTGCCAACATCTCCACCTGGGTCTACAGTAGCCCTGCTCGAGCATG  239
```

Figure S7. Agarose gel for quality control of *TPP1* minigene quantitative real-time PCR, and sequencing of PCR products. After completion of 40 cycles of quantitative real-time PCR, the samples shown in Suppl. Figure S6 were separated on an agarose gel. All primer pairs resulted in one single band of the expected size. No unspecific products or primer dimers were detected. The DNA bands from the agarose gel were sequenced with pcDNA3-reverse primer. The alignment shows the comparison of the sequenced PCR product (lower row) with the *TPP1* reference sequence (NM_000391.4, upper row). Exon 6 is shown in green, exon 7 in yellow. Exons 6 and 7 are correctly fused to each other, demonstrating correct splicing of the minigene mRNA.

Supplementary Figure S8

a

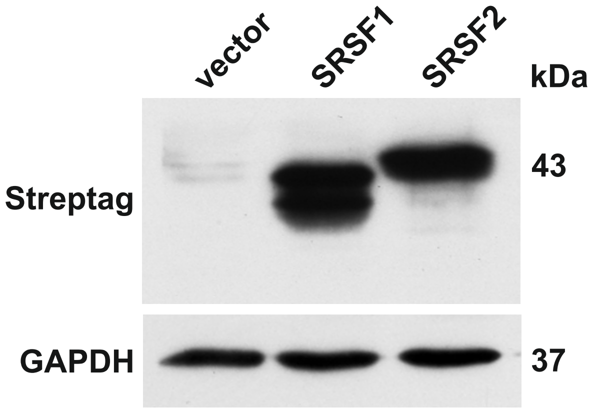

b

ATGGCGCGGAAGTCGAACTTGCCTGTGCTTCTCGTGCCGTTTCTGCTCTGCCAGGCCCTAGTGCGCTGCTCCAGC
CCTCTGCCCCTGGTCGTCAACACTTGGCCCTTTAAGAATGCAACCGAAGCAGCATTATTGGGATTTGATTAACGG
CGTGGAGGGCATTAGCATCTGGAGGCTCTGCCCTGGATGCAGTGGAGAGCGGCTGTGCCATGTGTGAGAGAGAGC
AGTGTGAC

Exon 1, 3' end of intron 1, mutation site, **exon 2**

Figure S8. Overexpression of Strep-tagged SRSF1 and SRSF2. HEK293T cells were cotransfected with equal amounts of the AGA minigene construct and an expression plasmid encoding the splicing factors SRSF1 or SRSF2. Both splicing factors are expressed with a C-terminal Streptag from the plasmid pEXPR-IBA103. Empty vector served as a control. (a) Expression of the splicing factors was confirmed by Western Blotting with an antibody specific for the Streptag. (b) Transfected cells were treated with luteolin for 24 h. Total RNA was isolated, reversely transcribed and used for PCR analysis with T7 forward vector primer and *AGA* exon 2 reverse primer. Sequencing of the dominant PCR product showed cryptic splicing of the *AGA* minigene transcript. Instead of a correct fusion of exon 1 and 2, the overexpression of splicing factors results in an aberrant splicing, i.e. inclusion of 23 bp from intron 1, irrespective of luteolin treatment.
